# Comparison of general anesthesia versus no anesthesia in elective transjugular intrahepatic portosystemic shunt (TIPS): Procedural and hemodynamic parameters

**DOI:** 10.1371/journal.pone.0341437

**Published:** 2026-02-10

**Authors:** Ziad Maksoud, Michael Köhler, Gala Nacul Mora, Florian Möllmann, Max Masthoff, Rainer Eßeling, Patrick Strauß, Arne Meier, Markus Kimmann, Jonel Trebicka, Michael Praktiknjo, Gesa Helen Pöhler

**Affiliations:** 1 Clinic for Radiology, University and University Hospital Münster, Albert-Schweitzer-Campus 1, Münster, Germany; 2 Department of Medicine B (Gastroenterology, Hepatology, Endocrinology and Infectious Diseases), University of Münster and University Hospital Münster, Albert-Schweitzer-Campus 1, Münster, Germany; Institute of Clinical and Experimental medicine: Institut klinicke a experimentalni mediciny, CZECHIA

## Abstract

Transjugular intrahepatic portosystemic shunt (TIPS) is an established treatment for complications of portal hypertension; yet the influence of anesthesia modality on procedural performance and hemodynamics remains insufficiently characterized. This retrospective single-center study compared radiation exposure, procedural parameters, and portal hemodynamics between procedures performed with and without general anesthesia (GA). A total of 84 patients were identified, of whom 62 were age- and sex-matched into two equal groups: group 1 (GA, n = 31) and group 2 (no GA, n = 31). Evaluated parameters included length of hospital stay, dose area product (DAP), fluoroscopy time (FT), contrast volume, number of digital subtraction angiography series, procedure duration, and pre- and post-TIPS measurements of portal venous pressure, central venous pressure (CVP), and portosystemic pressure gradient (PPG). Non-parametric statistical tests were applied. Patients in group 1 had significantly shorter postoperative hospital stay (median 6 days; interquartile range (IQR): 4–7) than those in group 2 (8 days; IQR 6–8; p = 0.006). Radiation dose was significantly lower in group 1, with a median DAP of 127.1 Gy*cm^2^ (IQR 64.6–201.8) compared to 325 Gy*cm^2^ (IQR 162.3–393.7; p = 0.02) in group 2. FT was also reduced under GA (12.2 minutes; IQR 9.6–15.9 vs. 16.0 minutes; IQR 11.5–25.9; p = 0.01), as was contrast volume (75 ml; IQR 60–100 vs. 90 mL; IQR 60–110; p = 0.01). PPG reduction was achieved in both groups, despite higher CVP under GA. These findings suggest that GA may facilitate more stable procedural conditions during TIPS, reducing radiation dose and contrast use without compromising hemodynamic effectiveness.

## 1. Introduction

Since the first placement in Germany approximately three decades ago [1], the transjugular intrahepatic portosystemic shunt (TIPS) has been subject to continuous evaluation and effort toward standardization in both its implementation and clinical application [2,3]. TIPS implantation is a complex angiographic procedure performed in selected patients with severe complications of portal hypertension [4]. Major parts of the procedure involve the use of fluoroscopy, digital subtraction angiography (DSA), administration of contrast agents, and precise hemodynamic pressure measurements [5].

The fluoroscopy time (FT), number of DSA runs, and volume of contrast agent used are influenced by the individual complexity of the case and the experience of the interventionalist. Despite advancements in TIPS technique and the recent practice guidelines by Cardiovascular and Interventional Radiological Society of Europe [3], there remains limited evidence regarding how different anesthesia modalities influence procedural parameters such as radiation dose and contrast agent consumption [6].

For elective TIPS procedures, the choice between general anesthesia (GA) or no anesthesia will depend on patient factors and local practice [7,8]. There is little literature comparing different anesthesia modalities and so the advantages and disadvantages must be considered for each individual case [7]. Most studies on anesthesia in the context of TIPS focus either on pre-procedural scoring systems to predict outcomes or on identifying the most suitable anesthetic agents for patients with liver disease [9]. However, there is limited evidence regarding the impact of anesthetic management on the technical aspects of the procedure itself.

At our institution, a shift in anesthesia protocol for TIPS was implemented in late 2014, transitioning from local anesthesia with optional sedation to GA.

This study aimed to compare radiation dose, procedural, and hemodynamic parameters during TIPS, under GA versus without GA.

## 2. Materials and methods

### 2.1. Study design and cohort

This retrospective study was conducted using anonymized clinical data. According to the Ethics Committee of the Medical Association Westfalen-Lippe and the University of Münster (reference number 2024–338-f-N), no formal ethical approval or informed consent was required.

A total of 84 patients who underwent elective, first-time TIPS placement between 01/01/2012 and 31/12/2016 at our tertiary center were retrospectively identified. Data were accessed for research purposes between 01/01/2025 and 30/09/2025.

Inclusion criteria were elective indication, performance by the same interventionist, standardized technique in the same angiography suite, access via the right internal jugular vein, transabdominal ultrasound-guided portal vein puncture, and absence of anatomical anomalies. Patients were excluded if TIPS was performed under emergency conditions (defined as variceal bleeding not controlled by endoscopic and pharmacologic therapy), if anatomical factors prevented standard access (e.g., portal vein thrombosis or variant venous anatomy), or if additional interventions or non-standard sedation were applied in the same session. In particular, patients receiving other sedation strategies such as total intravenous anesthesia without endotracheal intubation or analgosedation were excluded to ensure a homogeneous comparison between GA and local anesthesia.

**Fig 1:**
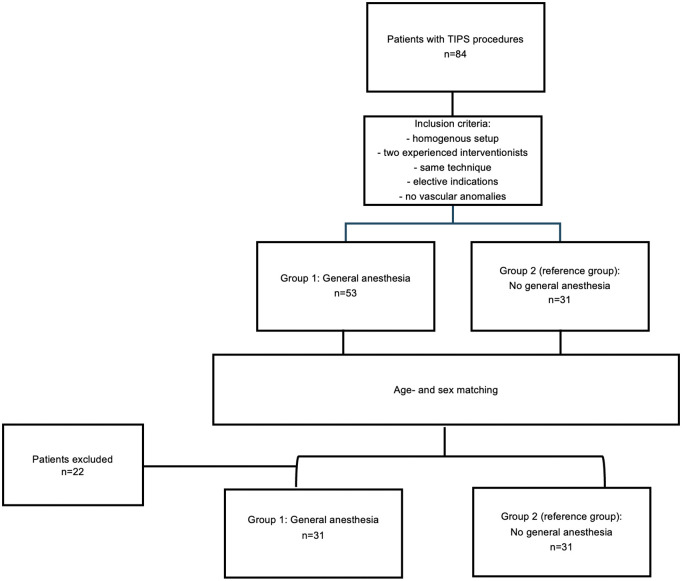
Flowchart of patients treated with Transjugular intrahepatic portosystemic shunt (TIPS) using different anesthesia procedures.

To create comparable study groups, patients were age- and sex-matched following initial eligibility assessment. This resulted in a final cohort of 62 patients, allocated to group 1 with GA (n = 31) and group 2 without GA (n = 31). The patient selection process is illustrated in (Fig 1).

### 2.2. Technique and angiography equipment

The same technique was used by the same two interventionists for all patients. The procedures were performed using a mono-planar angiography suite (Philips Healthcare, Allura FD 20, Netherlands; Siemens Healthineers, Artis icono ceiling) and the Rösch-Uchida Transjugular liver access set (Cook Medical, USA). For contrast, Ultravist 300 (Bayer, Germany) was used. All examinations were anonymized and transferred to a picture-archiving and communication system.

The right jugular vein was accessed under sonographic guidance. The access set and the wire (Amplatz Ultra-Stiff) were placed in the right hepatic vein using fluoroscopic control. Before portal vein puncture, a DSA was acquired. With sonographic guidance, the right portal vein was punctured. After central placing and pressure measurement, the stent graft (VIATORR®, W. L. Gore & Associates, Inc., USA) was deployed within the tract to establish a connection between the portal and hepatic vein and dilated by an 8 mm non-compliant balloon.

For all patients in group 1, the procedure was performed under GA with airway protection by an endotracheal tube. In collaboration with the anesthesiologist, ventilation was paused shortly while DSA series were acquired. Group 2 procedures were performed under local anesthesia only, without additional sedation.

None of the patients in either group underwent additional interventions, such as coiling or biopsy, ensuring that these factors did not contribute to higher radiation dose.

### 2.3. Parameters

#### 2.3.1. Clinical parameters.

Patient demographic information from the hospital information system was reviewed including sex, age, body mass index (BMI), Child-Pugh class, and the etiology of liver disease. The diagnosis of liver cirrhosis was based on a combination of clinical presentation, laboratory findings, and imaging features consistent with chronic liver disease and portal hypertension, in accordance with routine clinical practice.

#### 2.3.2. Radiation parameters.

The main outcome measures were the dose area product (DAP, in Gy*cm^2^) and FT (in minutes) logged in the radiology information system. Additionally, the number of DSA series was counted.

#### 2.3.3. Procedural and hemodynamic parameters.

Contrast agent volume (ml) was documented, and the procedure time was measured from the time of the first fluoroscopic acquisition to the final control DSA series. Pre- and post-TIPS central venous pressure (CVP), portal venous pressure (PVP), and the portosystemic pressure gradient (PPG) were recorded for each patient. CVP was measured via the introducer sheath positioned in the right atrium during the procedure. PVP was measured using the catheter positioned in the portal venous system. The PPG was calculated as the difference between PVP and CVP (PPG = PVP − CVP). Hepatic venous pressure gradient measurements were not performed, as the study focused on intra-procedural hemodynamics during TIPS creation.

In patients undergoing GA, both pre- and post-TIPS measurements were performed during controlled mechanical ventilation and prior to extubation.

### 2.4. Statistical analysis

All statistical analyses were performed using IBM SPSS Statistics version 29.0 (IBM Corp., Armonk, NY, USA). The normality of continuous variables was assessed using the Shapiro-Wilk test. Depending on data distribution, results are presented median with interquartile range (IQR) for non-normally distributed data. Categorical data are presented as absolute numbers and percentages. To compare categorical variables (e.g., sex, Child-Pugh class, liver disease etiology), we applied Fisher’s exact test or Fisher-Freeman-Halton test. For continuous variables, paired analyses were performed. The Wilcoxon signed-rank test was applied to compare matched groups, including radiation parameters (DAP, FT, and number of DSA series), procedural parameters (contrast agent volume, procedure time), hemodynamic pressures (CVP, PVP, and PPG), and length of hospital stay (days). Group distribution was compared using the Fisher–Freeman–Halton exact test. p-values < 0.05 were deemed statistically significant.

## 3. Results

### 3.1. Clinical parameters

Thirty-one of 62 patients were female (50%), and 31 male (50%). The median age was 61 (IQR 53–66.5) years with a median BMI of 25.3 (IQR 21.7–29.1). Child-Pugh class was determined as A for 10 patients (17%), as B for 41 patients (66%), and as C for 11 patients (17%). Refractory ascites was the leading indication for TIPS (85%), with similar distribution in group 1 (81%) and group 2 (90%); variceal hemorrhage was less common (19% vs. 10%; p = 0.22). Some patients had more than one cause of liver disease, so the main cause was also retrospectively not determinable. More details are demonstrated in Table 1.

**Table 1:** Baseline Demographics and Clinical Characteristics.

		All (n = 62)	Group 1 (n = 31)	Group 2 (n = 31)	p-value
**Sex, n (%)**					
	Male	31 (50)	21 (68)	21 (68)	0.27^1^
	Female	31 (50)	10 (32)	10 (32)	0.27^1^
**Age (years), median (IQR)**		61 (53–66.5)	62 (53–69)	60 (53–65)	0.2^3^
**BMI, median (IQR)**		25.3 (21.7–29.1)	25.3 (21.1–27.6)	25.3 (21.7–29.3)	0.3^3^
**Child-Pugh class, n (%)**					0.1^2^
	A	10 (16)	2 (6)	8 (26)	*
	B	41 (66)	22 (71)	19 (61)	*
	C	11 (18)	7 (23)	4 (13)	*
**Indication, n (%)**					0.2^3^
	Refractory ascites	53 (85)	25 (81)	28 (90)	*
	Variceal hemorrhage	9 (15)	6 (19)	3 (10)	*
**Cause of liver disease, n (%)**					
**hepatocellular**	Alcohol abuse	28 (45)	12 (38)	17 (55)	0.2^1^
	HBV	3 (5)	2 (6)	1 (3)	*
	HCV	3 (5)	1 (3)	2 (6)	*
	NASH	14 (22)	7 (23)	7 (23)	0.6^1^
	Cryptogenic	10 (16)	4 (12)	6 (19)	0.7^1^
	Autoimmune hepatitis	3 (5)	1 (3)	2 (3)	*
	Nutritional	6 (10)	2 (6)	4 (13)	*
**cholestatic**					
	PBC	4 (6)	4 (13)	0 (0)	*

Values are presented as numbers (%) or median and interquartile range (IQR), as appropriate. Fisher’s exact test^1^; Fisher-Freeman-Halton test^2^; Wilcoxon signed-rank teatabst^3^. Not applicable*. Abbreviations: BMI, body mass index; HBV, hepatitis B virus; HCV, hepatitis C virus; NASH, non-alcoholic steatohepatitis; PBC, primary biliary cholangitis.

Patients of group 1 had significantly shorter postoperative hospital stay (6 days; IQR 4–7) than patients of group 2 (8 days; IQR 6–8; p = 0.006).

### 3.2. Radiation parameters

The median DAP of group 1 (127.1 Gy*cm^2^; IQR 64.6–201.8) was lower than in group 2 (325 Gy*cm^2^; IQR 162.3–393.7; p = 0.002). Group 1 median FT was 12.2 minutes (IQR 9.6–15.9) and lower than in group 2 (16 minutes; IQR 11.5–25.9; p = 0.01). The number of DSA series was in group 1 (8; IQR 6–10) and did not differ significantly from group 2 (9; IQR 7–11; p = 0.3).

### 3.3. Procedural and hemodynamic parameters

**Fig 2:**
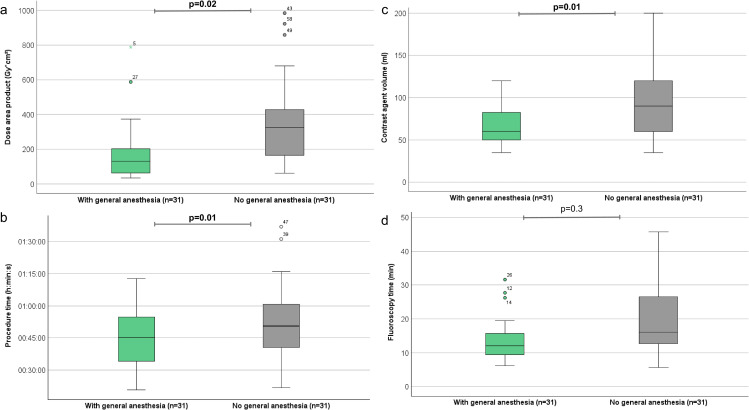
Graphical comparison of radiation and procedural parameters between group 1 (General anesthesia) and group 2 (No general anesthesia). **(a)** Dose area product (DAP, Gy·cm^2^) was significantly lower in group 1 (median 127.1; interquartile range [IQR] 64.6–201.8) compared to group 2 (325; IQR 162.3–393.7; p = 0.02). **(b)** Fluoroscopy time (FT, minutes) was significantly shorter in group 1 (median 12.2; IQR 9.6–15.9) than in group 2 (16; IQR 11.5–25.9; p = 0.01). **(c)** Contrast agent volume (ml) was significantly lower in group 1 (median 75; IQR 60–100) compared to group 2 (90, IQR 60–110; p = 0.01). **(d)** Total procedural time (minutes) did not differ significantly between groups.

The contrast agent volume was significantly lower in group 1 (75 ml; IQR 57–100) than in group 2 (90 ml; IQR 60–110; p = 0.01). The intervention time of group 1 (45.8 minutes; IQR 34.7–59.9) had no significant difference from group 2 (50.9 minutes; IQR 37.5–61.4; p = 0.4). More details are demonstrated in Table 2 and (Fig 2).

**Table 2:** Radiation parameters and procedural characteristics.

	All (n = 62)	Group 1 (n = 31)	Group 2 (n = 31)	p-value
**DAP (Gycm** ^**2**^ **), median (IQR)**	175.8 (100.1–3245.6)	127.1 (64.6–201.8)	325 (162.3–393.7)	0.02^1^
**FT (minutes), median (IQR)**	14.1 (10.6–19.3)	12.2 (9.6–15.9)	16 (11.5–25.9)	0.01^1^
**Procedural time (minutes), median (IQR)**	46.05 (35.9–60.7)	45.8 (34.7–59.9)	50.9 (37.5–61.4)	0.3^1^
**Number of digital subtraction angiography series, median (IQR)**	8 (6–10)	8 (6–10)	9 (7–11)	0.3^1^
**Pre-TIPS**	7 (5–8)	6 (5–8)	7 (6–9)	*
**Post-TIPS**	1 (1–2)	1 (1–1)	2 (1–2)	*
**Contrast volume (mL), median (IQR)**	75 (57–100)	60 (50–85)	90 (60–110)	0.01^1^

Values are presented as median and interquartile range (IQR). Wilcoxon signed-rank test^1^. Not applicable*. Abbreviations: DAP, dose area product; FT, fluoroscopy time; TIPS, transjugular intrahepatic portosystemic shunt.

CVP differed significantly between the groups both before and after TIPS. Pre-TIPS CVP was higher in group 1 (7.5 mmHg; IQR 5.7–12.2) compared to group 2 (4 mmHg; IQR 2–9.5; p = 0.004). Post-TIPS CVP remained higher in group 1 (11 mmHg; IQR 8–14.0) than group 2 (7 mmHg; IQR 4–10.5; p = 0.009). PVP did not differ significantly between groups. However, the resulting PPG was consistently lower in the GA group. Pre-TIPS PPG measured 15.5 mmHg (IQR 15–22) in group 1 vs. 21 mmHg (IQR 19–24.2) in group 2 (p = 0.005). Post-TIPS PPG was 7.5 mmHg (IQR 5.7–10.0) versus 10 mmHg (IQR 8.0–13.0), respectively (p = 0.02). The hemodynamic data are summarized in Table 3.

**Table 3:** Hemodynamic Measurements.

[all pressure values in mmHg]		All (n = 62)	Group 1 (n = 31)	Group 2 (n = 31)	p-value
**PPG reduction, median (IQR)**		10 (15–23.2)	9 (6.7–13.2)	11 (7.5–12.2)	0.3
**Pre-TIPS, median (IQR)**					
	CVP	7 (3–11)	7,5 (5.7–12.2)	4 (2–9.5)	0.004
	PVP	25 (21–31.5)	23 (21–30.5)	28 (21.5–32)	0.2
	PPG	19.5 (15–23.2)	15.5 (13–22)	21 (19–24.2)	0.005
**Post-TIPS, median (IQR)**					
	CVP	10 (6–13)	11 (8–14)	7 (4–10.5)	0.009
	PVP	18 (15–22)	18.5 (15–22.7)	18 (14.5–20.5)	0.3
	PPG	9 (11–7)	7.5 (5.7–10)	10 (8–13)	0.02

Hemodynamic parameters are presented as median and interquartile range (IQR). All intergroup comparisons were conducted using the Wilcoxon signed-rank test. Abbreviations: CVP = central venous pressure; PPG, portosystemic pressure gradient; PVP = portal venous pressure; TIPS, transjugular intrahepatic portosystemic shunt.

## 4. Discussion

This study compared elective TIPS procedures performed under GA with those performed with local anesthesia to evaluate potential differences in procedural characteristics and hemodynamic measurements. We observed a shorter hospital stay, lower radiation dose, shorter FT, and reduced contrast volume in the group treated under GA. In both groups, a reduction of the PPG to clinically effective levels was achieved, although CVP was higher under GA, consistent with the influence of controlled ventilation. These findings indicate measurable procedural differences associated with anesthesia modality while demonstrating that effective portal decompression can be obtained regardless of anesthesia approach.

The Cardiovascular and Interventional Radiological Society of Europe standards of practice emphasize the importance of procedural standardization to improve technical success and patient safety [3]. This approach represents the first comprehensive homogeneous study investigating anesthesia effects on TIPS procedural parameters under standardized technical conditions, enabling a focused evaluation of anesthesia-related impacts. By refining inclusion and exclusion criteria and subsequently matching patients into two comparable groups, we reduced heterogeneity and increased comparability.

Data on patient radiation dose during TIPS procedures remain limited, with only a few large series available for reference. Our study demonstrated lower DAP in TIPS procedures with GA. This finding aligns with previous studies, such as those by Miller et al. [10], that demonstrated a wide variability in radiation dose values. As a difference, our investigation specifically addresses the influence of anesthesia type while excluding major confounding factors representing a methodological advantage over earlier studies. Miller et al. [10] reported a 75th percentile DAP of 525 Gy·cm^2^ and a 75th percentile FT of 60 minutes across 134 TIPS procedures. In contrast, Miraglia et al. [2], using real-time ultrasound guidance for portal vein access in 211 TIPS cases, achieved a substantially lower 75th percentile DAP of 150 Gy·cm^2^ and an FT of 25.7 minutes. Bundy et al. [11] reported a 75th percentile DAP of 609 Gy·cm^2^ and an FT of 63.7 minutes in a cohort of 120 TIPS procedures. These findings highlight the variability in radiation exposure across centers and suggest that the use of ultrasound-guided techniques may play a critical role in reducing radiation burden. In line with previous findings [2,12,13], our study also employed real-time ultrasound-guided portal vein access during TIPS placement, which likely contributed to the overall procedural efficiency and supported favorable outcomes in terms of radiation dose and technical success.

Since ascites was the predominant indication for TIPS in our cohort, a potential influence on imaging conditions and radiation dose cannot be entirely excluded. Large-volume ascites may impair fluoroscopic visibility, necessitate repeated imaging runs, or prolong catheter manipulation, all of which could increase radiation dose [14]. Conversely, controlled conditions under GA may mitigate some of these effects by reducing patient movement and facilitating more stable imaging. As the amount of ascites was not systematically recorded in our study, we cannot quantify its contribution.

**Fig 3:**
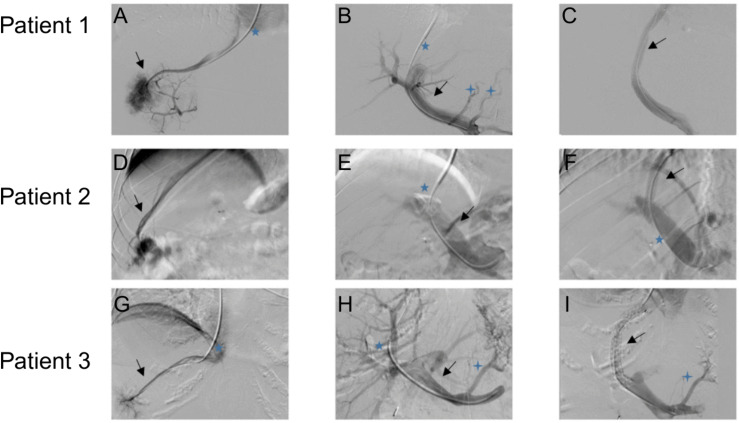
Comparison of transjugular intrahepatic portosystemic shunt (TIPS) placement under general anesthesia (GA) and local anesthesia. (**A–C)** Digital subtraction angiography (DSA) series acquired during TIPS placement under GA. **(A)** DSA with a 5F multipurpose catheter (black arrow) positioned in the right hepatic vein. **(B)** DSA with a 5F multipurpose catheter positioned in the splenic vein (blue star); the portal vein (black arrow) and portocaval anastomosis (4-headed stars) are visualized. **(C)** DSA after stent placement within the TIPS tract (black arrow). **(D-F)** DSA series acquired during TIPS placement under local anesthesia. **(D)** DSA with a 5F diagnostic catheter (black arrow) positioned in the right hepatic vein. **(E)** DSA with a 5F catheter (blue star) in the splenic vein; the portal vein is also visualized (black star). **(F)** DSA after stent placement within the TIPS tract (black arrow); the blue star demonstrates a 5F catheter positioned in the splenic vein. **(G-I)** DSA series acquired during TIPS placement under local anesthesia. **(G)** DSA with a 5F multipurpose catheter (black arrow) positioned in the right hepatic vein. **(H)** DSA with a 5F catheter positioned in the splenic vein (black arrow); the portal vein (blue star) and portocaval anastomosis (4-headed star) are also visualized. **(I)** DSA after stent placement (black arrow); portocaval anastomosis (4-headed star) remains visible. Respiratory motion is visible in selected images acquired without GA.

Evidence from other interventional fields suggests a potential influence of anesthesia on procedural efficiency and radiation dose. For instance, a study on endoscopic submucosal dissection reported reduced patient movement and shorter procedure times under GA compared to sedation, which often required airway management interruptions and prolonged the intervention [15]. Similarly, in neurointerventional radiology, anesthesia modality has been shown to affect FT and consequently radiation dose [16]. In our cohort, we also observed a difference in FT, whereas overall procedural duration did not show significant differences between the two groups. Although direct data on the influence of anesthesia type during TIPS creation are currently lacking, evidence from electrophysiological ablation procedures suggests that GA may improve procedural efficiency by enhancing catheter stability, thereby reducing both fluoroscopy and overall procedure times [17]. Additionally, findings from endovascular stroke interventions demonstrate that conscious sedation can be associated with increased patient movement and suboptimal image quality, potentially leading to repeated imaging, higher radiation dose, and increased contrast media usage [18,19]. These insights support the hypothesis that GA may confer similar advantages during TIPS procedures. The reduced radiation dose observed in the GA group can be attributed to several factors, notably the use of controlled respiratory maneuvers, which minimize patient movement and respiratory motion artifacts [20] (Fig 3).

Perioperatively, significantly lower contrast volume was utilized in group 1 patients (60 vs 90 ml). While the underlying reasons cannot be determined from our data, this difference may be related to more controlled procedural conditions under GA.

The CVP changes observed between the two patient groups undergoing TIPS in our study aligns with findings reported by Ushinsky et al. [9], who demonstrated that GA significantly alters intrathoracic and CVP conditions. In our cohort, a significant difference in pre- and post-TIPS CVP was noted between the two groups, with group 1 exhibiting a higher mean CVP. Despite these differences in CVP, both groups achieved post-TIPS PPG values within the clinically accepted therapeutic range (<12 mmHg), indicating effective portal decompression irrespective of anesthesia modality. The influence of mechanical ventilation on intra-procedural pressure measurements should therefore be considered when interpreting post-TIPS hemodynamic values obtained under GA. Consequently, the observed reductions in radiation dose, FT, and contrast use under GA in our study are unlikely to be attributable solely to hemodynamic variation, but rather to improved procedural conditions such as reduced patient motion and greater catheter stability under controlled ventilation.

Our study has some limitations. Due to the retrospective nature, there is less influence on the recorded information. The change in anesthesia protocol and angiography suite over the study period may also have influenced the results. Procedure time was defined only as the interventional time and did not include the overall setting. Anesthesia-related complications, prior episodes of hepatic decompensation, and patient-related factors such as the amount of ascites, respiratory motion, or procedural restlessness were not systematically assessed. As ascites volume and intra-abdominal pressure were not recorded, imaging conditions and intra-procedural portal pressure measurements may have been affected; however, this limitation applies to both groups, as refractory ascites was the predominant indication for TIPS. Finally, post-TIPS pressure measurements in patients undergoing GA were obtained under controlled mechanical ventilation prior to extubation, which should be considered when interpreting hemodynamic values.

## 5. Conclusion

In this systematically evaluated age- and sex-matched TIPS cohort, GA was associated with lower radiation dose, shorter FT, reduced contrast use, and a shorter length of hospital stay, while maintaining effective portal decompression in both groups. The differences in CVP observed under GA align with expected physiological effects of controlled ventilation and did not alter the therapeutic reduction of the PPG. While GA may support more stable procedural conditions and thereby improve efficiency, its use also requires greater logistics, staff coordination, and resource allocation. Importantly, TIPS without GA remained clinically effective and hemodynamically successful in this cohort. Therefore, anesthesia modality should be selected based on procedural complexity, patient condition, and institutional resources. Prospective studies are warranted to further evaluate clinical and workflow-related implications.
